# Mild Salt Stress Conditions Induce Different Responses in Root Hydraulic Conductivity of *Phaseolus vulgaris* Over-Time

**DOI:** 10.1371/journal.pone.0090631

**Published:** 2014-03-04

**Authors:** Monica Calvo-Polanco, Beatriz Sánchez-Romera, Ricardo Aroca

**Affiliations:** Estación Experimental del Zaidín (CSIC), Department of Soil Microbiology and Symbiotic Systems, Granada, Spain; University of Vigo, Spain

## Abstract

Plants respond to salinity by altering their physiological parameters in order to maintain their water balance. The reduction in root hydraulic conductivity is one of the first responses of plants to the presence of salt in order to minimize water stress. Although its regulation has been commonly attributed to aquaporins activity, osmotic adjustment and the toxic effect of Na^+^ and Cl^−^ have also a main role in the whole process. We studied the effects of 30 mM NaCl on *Phaseolus vulgaris* plants after 9 days and found different responses in root hydraulic conductivity over-time. An initial and final reduction of root hydraulic conductivity, stomatal conductance, and leaf water potential in response to NaCl was attributed to an initial osmotic shock after 1 day of treatment, and to the initial symptoms of salt accumulation within the plant tissues after 9 days of treatment. After 6 days of NaCl treatment, the increase in root hydraulic conductivity to the levels of control plants was accompanied by an increase in root fructose content, and with the intracellular localization of root plasma membrane aquaporins (PIP) to cortex cells close to the epidermis and to cells surrounding xylem vessels. Thus, the different responses of bean plants to mild salt stress over time may be connected with root fructose accumulation, and intracellular localization of PIP aquaporins.

## Introduction

Soil salinity is a major problem in many areas around the world as it affects plant establishment, development and productivity [1]. Plants are primarily affected by salt due to its osmotic effect since the osmotic water potential of the soil decreases with increasing salt concentration [2]. Plants respond to the reduced water availability in the soil by reducing their leaf transpiration [3], stomatal conductance [4], and by adjusting their root water uptake [5]. Plants also face ion toxicity, mainly due to Na^+^ and Cl^−^ accumulation [6]–[8], which disturbs major plant metabolic processes, such as enzyme activity, lipid metabolism and protein synthesis [9]–[11].

The main resistance to water transport in roots resides in the radial pathway, which consists of apoplastic and cell-to-cell water pathways (symplastic and the transmembrane) [12]. The regulation of root hydraulic conductivity (L) in plants under stress has been mainly attributed to cell-to-cell pathway and in particular to the expression and abundance of transmembrane aquaporins, with the apoplastic water flow limited by the presence of suberized barriers as the exo- and/or the endodermis [12], [13] and Casparian strips [14]. Many studies dealing with the effect of NaCl in root hydraulic properties have showed a decrease in L, however, most of them have focused on relative short periods of time (from minutes up to 24 hours) [15]–[18]. Under prolonged periods of exposure to salt, a partial recovery of L could take place [19], and this could be mainly caused by the accumulation of compatible solutes within the plant roots [5].

Aquaporins are intrinsic membrane proteins which are present in all living organisms [20] and which facilitate the transport of water and other small uncharged molecules across cell membranes along a concentration gradient [21]–[23]. Plant aquaporins are classified into several subfamilies [24], [25] with the plasma membrane intrinsic proteins (PIPs) thought to be responsible for the regulation of L [26], [27]. Although it is well known that NaCl is a inhibitor of aquaporin-mediated root water transport [28], [29], the regulation of L under salt stress has been shown to be directly correlated with the PIP gene expression [30], [31], as well as with protein phosphorylation and dephosphorylation events [17].

Plants may also accumulate metabolites under stress to balance their cytoplasm water potential and to prevent water moving from the roots to the soil [32]. Plants accumulate inorganic and organic compounds such as proline, glycine, betaine, soluble sugars, pinitol and mannitol within the cell cytoplasm without interfering with cell metabolism [33], [9]. Sugars also participate in the synthesis of other compounds, the production of energy, membrane stabilization [33], regulation of genes expression [34], and as signal molecules [35]. Although it has been demonstrated that the accumulation of soluble sugars in the plant tissues is a crucial factor for plant salt tolerance [36], the studies up to date have mainly concentrated on the role of glucose and sucrose. Also, the knowledge of the role of fructose in water relations is limited, with just one study showing a direct relationship between the presence of fructose and an increase on root exudation rates in sunflower plants [37].

The main objective of this study was to determine if the exposure of *P. vulgaris* plants to a relatively low NaCl concentration of 30 mM for 9 days would alter L through the effect of salt on aquaporins expression, abundance and phosphorylation status, as well as root sugars concentrations and the toxic effect of Na^+^ and Cl^−^ accumulation. We hypothesized that (1) the plants would acclimate to NaCl over time based on the increase of L, (2) the presence of osmolytes would affect the Lof the plants, which combined with the aquaporins function, will maintain a fine water balance under salt stress, and (3) prolonged exposure to NaCl would cause toxic effects in plants which, in turn, would cause a reduction L.

## Materials and Methods

### Plant material and growth conditions

Seeds of *Phaseolus vulgaris* (cultivar Borlotto) plants were germinated in wet perlite for one week and then transferred into an aerated mineral solution at 80% [38]. The plants were grown for another ten days before the start of the treatments in a controlled-environment growth chamber at 22/18°C, 60% relative humidity, 18/6 day:night photoperiod and a photosynthetic photon flux density of 300–350 µmol m^−2^ s^−1^. The solutions were changed every third day during the length of the experiment.

The plants were subjected to 0 and 30 mM NaCl for 9 days. In a first experiment, osmotic root hydraulic conductivity was determined after 0, 1 and 6 hours and after 1, 3, 6 and 9 days from the start of the treatments. Based on these results, we chose day 1, 6 and 9 as the base for the determination of the different physiological and molecular parameters present in this study. The experiment was independently repeated three times with similar results. The plants measured at the different days were of the same age and have the same developmental stage. After 9 days of treatment, the plant had not set flowers.

From the results of the previous experiment, we saw a recovery of L after six days of NaCl treatment that was correlated with an increase on root fructose content. As the information of the role of fructose on L is limited, we tested whether the presence of fructose could increase L. We treated plants with 0 (Control), 30 mM NaCl, 3 mM fructose, and 3 mM fructose +30 mM NaCl for 9 days and determined their osmotic root hydraulic conductivity at day 1, 3, 6 and 9.

### Stomatal conductance (gs), leaf water potential and plant dry weights

Stomatal conductance and dry weights were determined in six plants after 1, 6 and 9 days of NaCl treatment (n = 6). Stomatal conductance was measured in fully grown mature leaves with a portable AP4 Porometer (Delta-T Devices Ltd, Cambridge) four hours after the lights were on within the growth chamber.

Leaf water potential was determined with a pressure chamber (SF-PRES-35, SolFranc Tecnologías SL, Tarragona) on six mature leaves after 1, 6 and 9 days of NaCl treatment (n = 6). Mature fully developed leaves were excised from the main shoot after the *gs* measurements and introduced into the pressure chamber. Pressure was applied until xylem sap was visible at the cut surface.

Plant shoot, root and total dry weights were determined in 6 plants (n = 6) per NaCl treatment at day 1, 6 and 9, after oven-dry of the plants tissues at 65°C for 48 hours.

### Root hydraulic conductivity (L)

Root hydraulic conductivity was measured by the free-exuded sap method after 0, 1 and 6 h, and after 1, 3, 6 and 9 days in plants treated with 0 and 30 mM NaCl. The results were contrasted with the measurement of L with a pressure chamber after 1, 3, 6 and 9 days of NaCl treatment. Also, L was measured with the free-exudated sap method in plants treated with fructose and NaCl after 1, 3, 6 and 9 days of treatment.

Osmotic root hydraulic conductivity (L_o_) was calculated in six detached roots (n = 6) four hours after the lights were on (time 0) within the grow chamber. For days 1, 3, 6 and 9, L_o_ was measured at the same time of the day (four hours after lights turned on). Plants were cut below the cotyledons and the exudates collected with a silicone tube for 3 hours (time to allow enough amount of exudation in the NaCl treatments to be analyzed). The exudates collected for the fifteen first minutes were discarded to avoid phloem contamination. A cryoscopic osmometer (Osmomat 030, Gonotec Gmbh, Berlin) was used to determine the osmolarity of the exuded sap and the nutrient solution. Osmotic L was calculated as L_o_ = J_v_/Δψ_s_, were J_v_ is the exuded sap flow rate per hour and unit of root dry weight and Δψ_s_ the osmotic potential difference between the nutrient solution and the exuded sap.

Root hydraulic conductivity was also measured with a pressure chamber (L_p_) (SF-PRES-35, SolFranc Tecnologías SL, Tarragona) in six roots (n = 6) per treatment 4 hours after the lights were on within the grow chamber (the same time range than for L_o_). When detached roots were placed into a pressure chamber (filled with the same nutrient solution *plus*/*minus* 30 mM NaCl), the root flow rate was stabilized for 5 minutes and plants exudates were collected for another 5 min at hydrostatic pressures of 0.1, 0.3 and 0.5 MPa. Root hydraulic conductance (K_r_) was obtained from the regression line of flow rates plotted against hydrostatic pressures. The K_r_ values were divided by root dry weights to obtain root hydraulic conductivity (L_p_).

### Root aquaporin-PIP expression analyses

Aquaporin PIP expression was determined in roots of three plants per treatment after 1, 6 and 9 days. Total RNA was isolated by the phenol/chloroform extraction method followed by LiCl precipitation [39]. DNase treatment of total RNA and reverse transcription were done following Qiagen's protocol (Quantitect Reverse Transcription KIT Cat#205311, Qiagen, CA). The analyses of aquaporin expression were done for the six PIP genes described so far in *P. vulgaris*, *PvPIP1;1* (Acc. No. U97023), *PvPIP1;2* (Acc. No. AY995196), *PvPIP1;3* (Acc. No. DQ855475), *PvPIP2;1* (Acc. No. AY995195), *PvPIP2;2* (Acc. No. EF624001), and *PvPIP2;3* (Acc. No. EF624002) as described in Benabdellah et al. [40]. The expression of the different aquaporins was determined using a real time quantitative PCR (iCycler-Bio-Rad, Hercules, CA). Each 23 µl reaction mixture contained 1 µl of cDNA, 10.5 µl of Master Mix (Bio-Rad Laboratories S.A, Madrid), 8.6 µl of deionized water, and 0.45 µl of each primer pair at a final concentration of 0.2 µM. The PCR program consisted in 3 min incubation at 95°C, followed by 32 cycles of: 30 s at 95°C, 30 s at 60°C of annealing temperature for *PvPIP1;2* and *PvPIP2;2*, and 30 s at 58°C of annealing temperature for *PvPIP1;1*, *PvPIP1;3, PvPIP2;1* and *PvPIP2;3*, and 72°C for 30s. The specificity of the PCR amplification procedure was checked with a heat dissociation protocol (from 60 to100°C) after the final cycle of the PCR. The relative abundances of transcription were calculated using the 2-^ΔΔ^ *C*_t_ method [41]. Ubiquitin-specific primers were used for standardization by measuring the expression of the *P. vulgaris* ubiquitin gene in each sample [42]. Ubiquitin was chosen as it expression was stable at all treatments and days considered (NaCl C_t_ 24.3±0.3 (mean ± SE); Control C_t_ 24.7±0.2 (mean ± SE)). Three different root RNA samples from the salt treatment and the different days of measurement were used for the analysis (n = 3), with each of them repeated three times. Negative controls without cDNA were used in all the PCR reactions.

### Microsomal preparation, ELISA analyses and western blots

Microsomes were isolated after 1, 6 and 9 days of treatment as described in Hachez et al. [43] with some modifications. About 50 mg of fresh-frozen roots were homogenized with 6 ml of grinding buffer: 250 mM sorbitol; 50 mM Tris–HCl pH = 8; 2 mM EDTA; and proteinase inhibitors (1 mM phenylmethylsulfonyl fluoride, and 1 µg ml^−1^ of leupeptin, aprotinin, antipain, chymostatin and pepstatin). The mixture was filtered with a nylon mesh and centrifuged at 4,400 g for 10 min. The supernatant was then centrifuged at 100,000 g for 2 h. The resulting pellet was resuspended in 20 µl of 5 mM KH_2_PO_4_, 330 mM sucrose, and 3 mM KCl with a final pH of 7.8.

Two micrograms of protein extracts were used for ELISA analyses. The extracts were incubated in Immulon 4HBX microplates (Thermo Fisher Scientific Inc., Belgium) at 4°C for 24 hours in carbonate/bicarbonate coating buffer at pH = 9.6. The following day, the wells were cleaned with 3×10 min washes using Tween Tris-buffered saline solution (TTBS), and blocked with 1% bovine serum albumin (BSA) on TTBS for 30 min at room temperature. After another 3×10 min washes with TTBS, proteins were incubated with 100 µl of the primary antibody (1∶2000 on TTBS, v/v) for 1 hour at room temperature. We used, as primary antibodies, the two antibodies that recognize several PIP1 and PIP2 proteins [30] and three antibodies that recognize the phosphorylation of PIP2 proteins at their C-terminal region. All the antibodies were designed against the most conservative regions of these aquaporin groups. To detect PIP1 aquaporins, we used the first 26 aa of the N-terminal part of *PvPIP1;3* protein (Acc. No. DQ855475, [30]), as a peptide to immunize rats. To detect PIP2 aquaporins, we used the last 12 aa of the C-terminal part of *PvPIP2;1* protein (Acc. No. AY995195, [44]) as a peptide to immunize rabbits. To detect phosphorylated PIP2, we used the same protein *PvPIP2;1* as amino acid sequence but with the different serine groups phosphorylated as follows: PIP2A (Ser-280), AIKLALG{pSER}FRSNA; PIP2B (Ser-283), AIKALGSFRS{pSER}NA and PIP2C (Ser-280 and Ser-283), AIKALG{pSER}FR{pSER}NAC (Abyntek Biofarma SL, BiotechSpain, Vizcaya). A goat anti-rat Ig coupled to horseradish peroxidase (Sigma-Aldrich Co., USA) antibody was used at a secondary antibody at 1∶10000 for PIP1. Goat anti-rabbit Ig coupled to horseradish peroxidase (Sigma-Aldrich Co., USA) was used as a secondary antibody at 1∶10000 for PIP2 and PIP2A, B and C. Protein quantification was done in three different independent root samples per salt treatment and day of measurement (n = 3), repeated three times each. PIP1 and PIP2 antibodies antigens were aligned to see if they could recognize other *P.vulgaris* aquaporins (Table S1). To determine the specificity of the PIP2 and phosphorylated antibodies PIP2A, B, and C, we ran ELISA analysis with 2 µg of pure peptides PIP2, PIP2A, PIP2B, PIP2C, crossed each of them with all the PIP2 antibodies. ELISA analysis of pure peptides was conducted as described above for the roots protein extracts (Table S2).

Western blots were developed to determine whether antibodies against phosphorylated PIP2A, B and C aquaporins, could recognize the same bands as the antibody against non-phosphorylated PIP2 aquaporins. Microsomes were isolated as described above and ten micrograms of protein were loaded in each line of a 12% Amersham ECL gel (GE Healthcare Bio-Sciences, Upsala), after incubating for 15 min at 60°C in the presence of denaturing buffer (20 mM Tris–HCl pH = 8.6, 1% (w/v) SDS, 0.3% (v/v) *b*-mercaptoethanol, 8% (v/v) glycerol, 0.2% (w/v) bromophenol-blue). Proteins were transferred to a PVDF membrane at 1.3 A for 7 min (Trans-Blot Turbo Transfer System, Bio-Rad Laboratories SA, Madrid). The membrane was blocked for 2 h at room temperature with 5% (w/v) non-fat milk in Tris-buffered-saline (TBS) with 0.05% Tween 20. After that, the membrane was incubated overnight at 4°C with the PIP2 and phosphorylated PIP2 antibodies at 1∶1000. Goat anti-rabbit Ig coupled to horseradish peroxidise (Sigma-Aldrich Co., USA) was used as a secondary antibody at a dilution of 1∶10000. The signal was developed using a chemiluminescent substrate (West-Pico,Super Signal, Pierce, Rockford, IL, USA). Western blots were developed from two different sets of microsomes with no significant differences between them (Figure S1).

### Root PIP immunolocalization and suberization of cortical layers

Roots from four control and four NaCl treated plants (n = 4) were selected after 1, 6, and 9 days of NaCl treatment. Fresh free-hand sections were obtained with a razor blade at 5–10 mm distance from the root tip. For immunolocalizaton of aquaporins, we followed the procedure described in Hachez et al. [43] with two primary antibodies and negative controls. We used the same antibodies described at the ELISA procedure, with primary antibodies PIP1 and PIP2 applied at 1∶2000 (v/v), and secondary antibodies applied at 1∶1000 (v/v). Fluorescein-coupled donkey anti-rat IgG antibody was used as a secondary antibody for the PIP1 (H&L, DyLight 488 conjugated, Agrisera AB, Sweden), and fluorescein-couple goat anti-rabbit IgG antibody for the PIP2 (H&L, DyLight 549 conjugated, Agrisera AB, Sweden). The slides were examined under a fluorescence microscope using a green filter B-2A (Nikon Eclipse 50i, Nikon Instruments Europe BV, Badhoevedrop, NL) for PIP1 at 493 nm excitation and 518 nm emission. For the PIP2 sections we used a red filter G-2A (Nikon Eclipse 50i, Nikon Instruments Europe BV, Badhoevedrop, NL) at 562 nm excitation and 576 nm emission.

The accumulation of suberin and the formation of casparian bands at the exodermis and endodermis level were examined in roots of four control and four NaCl treated plants after 1, 6, and 9 days of NaCl treatment. Fresh free-hand sections were obtained with a razor blade at 5–10 mm distance from the root tip. The sections were stained with for 60 min with berberine hemisulphate, and counter-stained for 1 min with toluidine blue O [45]. The sections were examined under a fluorescence microscope using a green light filter B2-A at 470–490 nm excitation and 505 nm emission (Nikon Eclipse 50i, Nikon Instruments Europe BV, Badhoevedrop, NL).

### Root metabolites determination and damage

Root proline content and oxidative damage to lipids were determined in six plants per treatment on each measurement date (n = 6). Roots (50 mg fresh weight, FW) were extracted with 5% sulphosalicylic acid [46]. Proline was determined by a colorimetric method using ninhydrin as a reagent [47]. Root oxidative damage to lipids was determined as described in Ruiz-Lozano et al. [48]. Proline and the oxidative damage to lipids were quantified with a spectrophotometer (Infine 200 PRO NanoQuant, Tecan Ibérica Instrumentación SL, Barcelona) at 532 nm.

Root electrolyte leakage was measured in six complete root systems per salt treatment and day of measurement (n = 6) as described by Aroca et al. [49].

Root glucose, fructose and sucrose tissue concentrations were determined in three plants per salt treatment and measurements date (n = 3). Roots (50 mg FW) were extracted in water at 60°C for 1 h and extracts were filtered with a 40 µm syringe filter. Glucose was determined in the extracts using the Glucose (GO) Assay Kit (GAGO-20, Sigma-Aldrich Co), fructose with the Fructose Assay Kit (FA-20, Sigma-Aldrich Co) and sucrose with the Sucrose Assay kit (SCA-20, Sigma-Aldrich Co). Resulting sample extracts were measured in a spectrophotometer a (HITACHI U-1900, Hitachi High-Technologies Corporation, Tokyo) at 540 nm for glucose and 340 nm for fructose and sucrose.

### Na^+^ and Cl^−^ tissue and xylem sap concentrations

Na^+^ and Cl^−^ concentrations from roots and from completely developed-mature leaves were determined in six plants after 1, 6 and 9 days of NaCl treatments (n = 6). For Na^+^ determination, 0.3 to 0.5 g of dry tissue was extracted with the HNO_3_/H_2_O_2_ method and the extracts were measured using an iCAP 6500 ICP Spectrometer (Thermo Fisher Scientific Inc., Belgium). For Cl^−^ determination, 0.01 g of dry tissue was extracted with 1.5 ml of hot water. The Cl^−^ concentrations in the extracts of the tissues were determined following the procedure described in Diatloff & Rengel [50] and using an Infinite 200 PRO Reader (TECAN Group Ldt., Switzerland).

Na^+^ and Cl^−^ xylem sap were measured in the exudates obtained for L_p_ in six plants (n = 6) per treatment combination at day 1, 6 and 9. Na^+^ was determined with an iCAP 6500 ICP Spectrometer (Thermo Fisher Scientific Inc., Belgium). Cl^−^ was determined following the same procedure described above.

### Statistical analysis

Data were analyzed using the MIXED Procedure in SAS (version 9.2, SAS Institute Inc., NC, USA). Two-way analysis of variance (ANOVA) was used to compare the control and salt treatments at the different days of measurement. For the graph that involves the L in the presence of fructose, we also compare all the treatments at the different days of measurement. Tukey's adjustment was used as the post-hoc test to detect significant differences between treatment means at α = 0.05.

## Results

### Stomatal conductance, leaf water potential and plant dry weights

Stomatal conductance significantly decreased in NaCl treated plants after 1 and 9 days of treatment compared with their respective control plants. Salt treated plants reached similar *gs* values as control plants after six days of treatment (Table 1). Leaf water potential did not change significantly in control plants over the experiment, but when NaCl was applied, it was significantly higher (less negative) after 6 days of treatment compared with plants treated for 1 and 9 days (Table 1).

**Table 1:** Physiological parameters.

	(+) 1 Day		(+) 6 Days		(+) 9 Days	
	Control	NaCl	Control	NaCl	Control	NaCl
Leaf *gs* (mmol m^−2^ s^−1^)	91.2±3.30a	69.0±4.89b	45.1±9.33bc	39.3±7.75cd	57.3±3.12bc	38.1±4.81d
Leaf ψ_w_ (MPa)	−0.35±0.04a	−0.54±0.04b	−0.27±0.05a	−0.33±0.04a	−0.27±0.04a	−0.46±0.04b
Shoot DW (g)	0.54±0.05d	0.51±0.05d	1.19±0.19c	1.26±0.10c	2.18±0.16a	1.64±0.13b
Root DW (g)	0.10±0.01c	0.11±0.01c	0.21±0.01b	0.22±0.02b	0.31±0.03a	0.24±0.02b
Total DW (g)	0.64±0.04d	0.62±0.05d	1.40±0.20c	1.48±0.12bc	2.49±0.18a	1.88±0.15b
Root Fructose (mg g^−1^ FW)	15.32±2.18a	3.46±0.59bc	1.67±1.28c	7.79±2.63b	4.43±2.41bc	8.37±0.31b
Root Glucose (mg g−1 FW)	2.62±0.32a	1.17±0.18b	1.38±0.12b	1.45±0.17b	1.46±0.08b	1.59±0.14b
Root Sucrose (mg g−1 FW)	4.27±0.62a	3.53±0.69a	3.03±0.56a	4.37±0.53a	3.56±0.98a	4.62±0.20a

Leaf stomatal conductance (*gs*), leaf water potential (ψ_w_), shoot, root and total dry weights (DW), and root fructose, glucose and sucrose content of *Phaseolus vulgaris* control non-treated plants and plants treated with 30 mM NaCl after 1, 6 and 9 days. Significant differences among treatment means at the different days of measurement are shown with different letters at α = 0.05. Means (n = 6) ± SE are shown.

Control and salt-treated plants did not show any statistically significant differences in shoot, root and total dry weights after 1 and 6 days of treatment, however there were significant reductions in the root, shoot and total dry weights of salt treated plants after 9 days (Table 1).

### Root hydraulic conductivity

**Figure 1:**
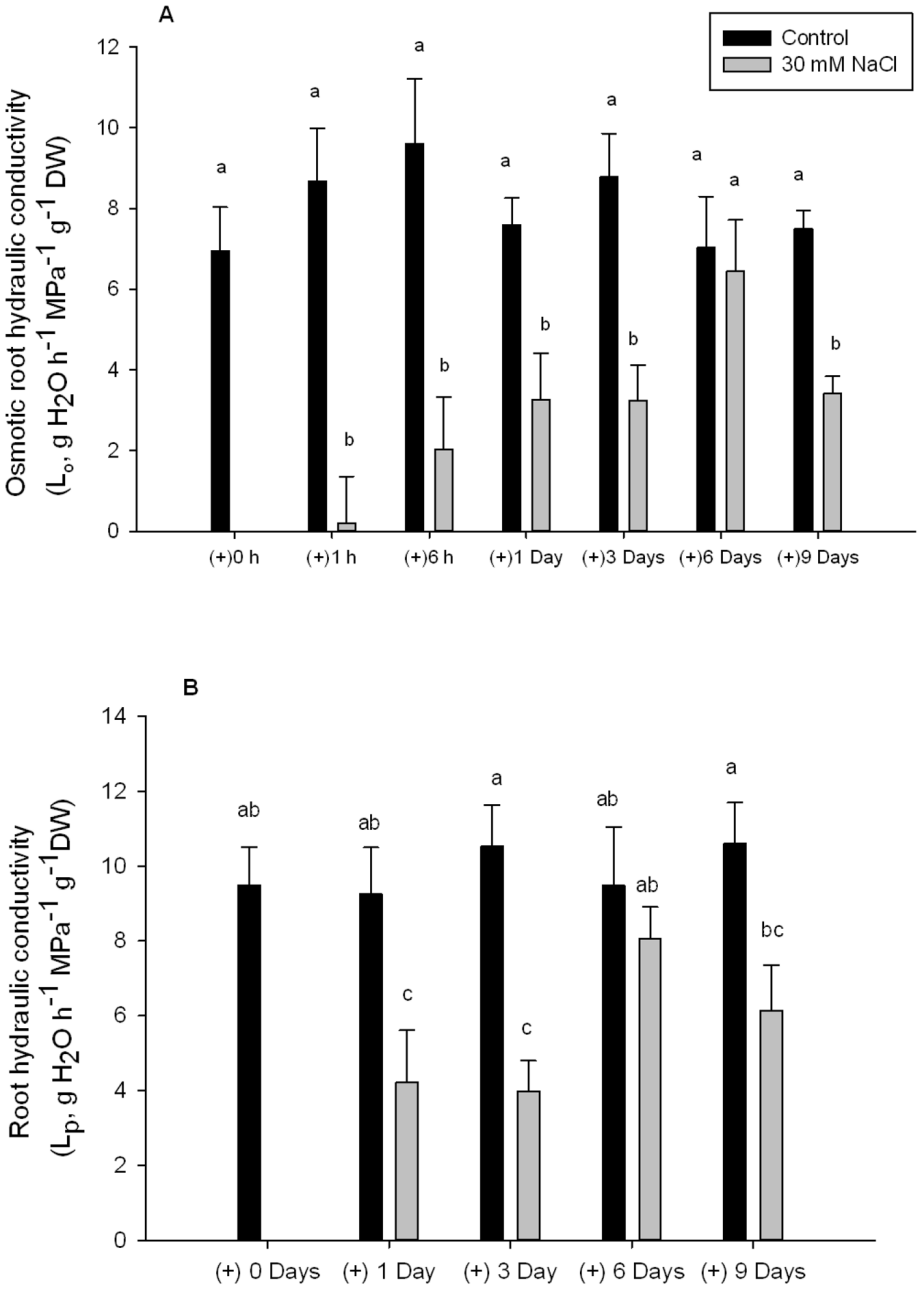
Root hydraulic conductivity. Root hydraulic conductivity in *Phaseolus vulgaris* treated with of 0 and 30 mM NaCl after 1, 3, 6 h and after 1, 3, 6 and 9 days determined by the exudates method (A) and after 1, 3, 6 and 9 days determined with a pressure chamber (B). Significant differences between treatment means at the different days of measurement are shown with different letters at α = 0.05. Means (n = 6) ± SE are shown.

Osmotic root hydraulic conductivity (L_o_) was significantly lower in NaCl treated plants compared with the respective controls for all measured times except for day 6, where they had the same L_o_ values as their respective control (Figure 1A). Root hydraulic conductivity measured with the pressure chamber was also significantly lower after 1 and 3 days of NaCl treatment compared with their controls. There was also a recovery of L_p_ to the values of control plants after 6 days of treatment, and again a L_p_ decrease after 9 days of NaCl treatment (Figure 1B).

### Root PIP expression, abundance and phospohrylation analyses

**Figure 2:**
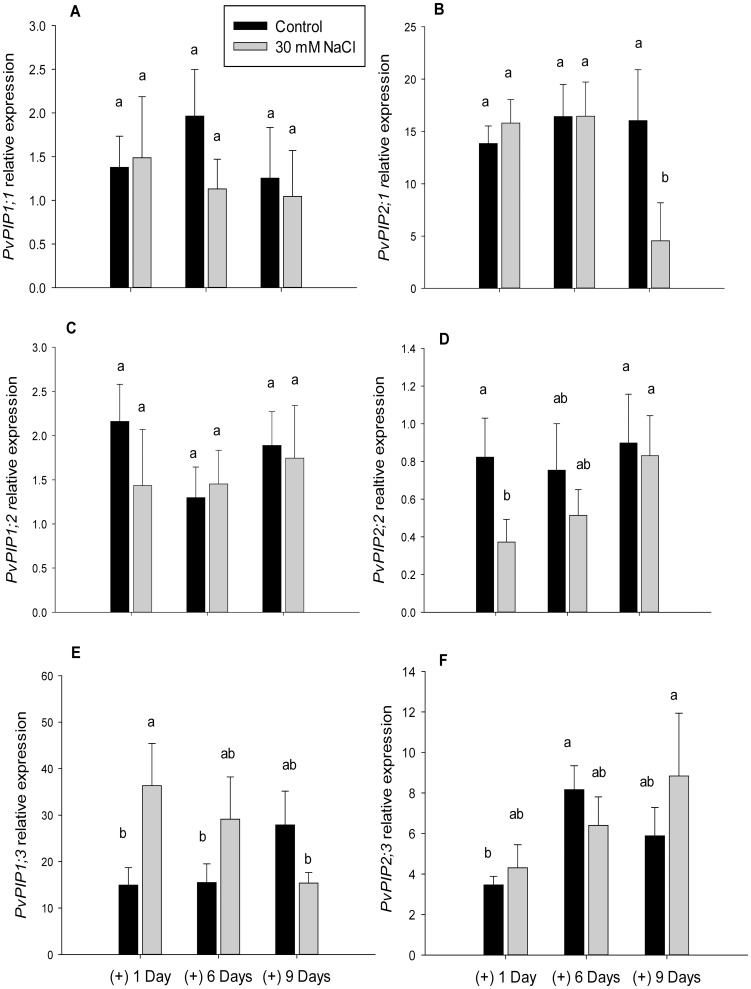
Aquaporin expression. *P.vulgaris* plants root aquaporin expression of *PvPIP1;1* (A), *PvPIP1;2* (C), *PvPIP1;3* (E), *PvPIP2;1* (B), *PvPIP2;2* (D), and *PvPIP2;3* (F), treated with 0 and 30 mM NaCl after 1, 6 and 9 days and expressed as relative units (r.u). Significant differences between treatment means at the different days of measurement are shown with different letters at α = 0.05. Means (n = 3) ± SE are shown.

Gene expression of *PvPIP1;1, PvPIP1;2* and *PvPIP2;3* of NaCl treated plants did not show significant differences with their control plants at any of the measured days (Figure 2A,C,F). After 1 day of treatment, the expression of *PvPIP1;3* in NaCl treated plants increased (Figure 2E), while the expression of *PvPIP2;2* showed a significant decrease at the same time point (Figure 2D). Finally, there was a decrease of the *PvPIP2;1* gene expression after 9 days of treatment in NaCl-treated plants (Figure 2B).

**Figure 3:**
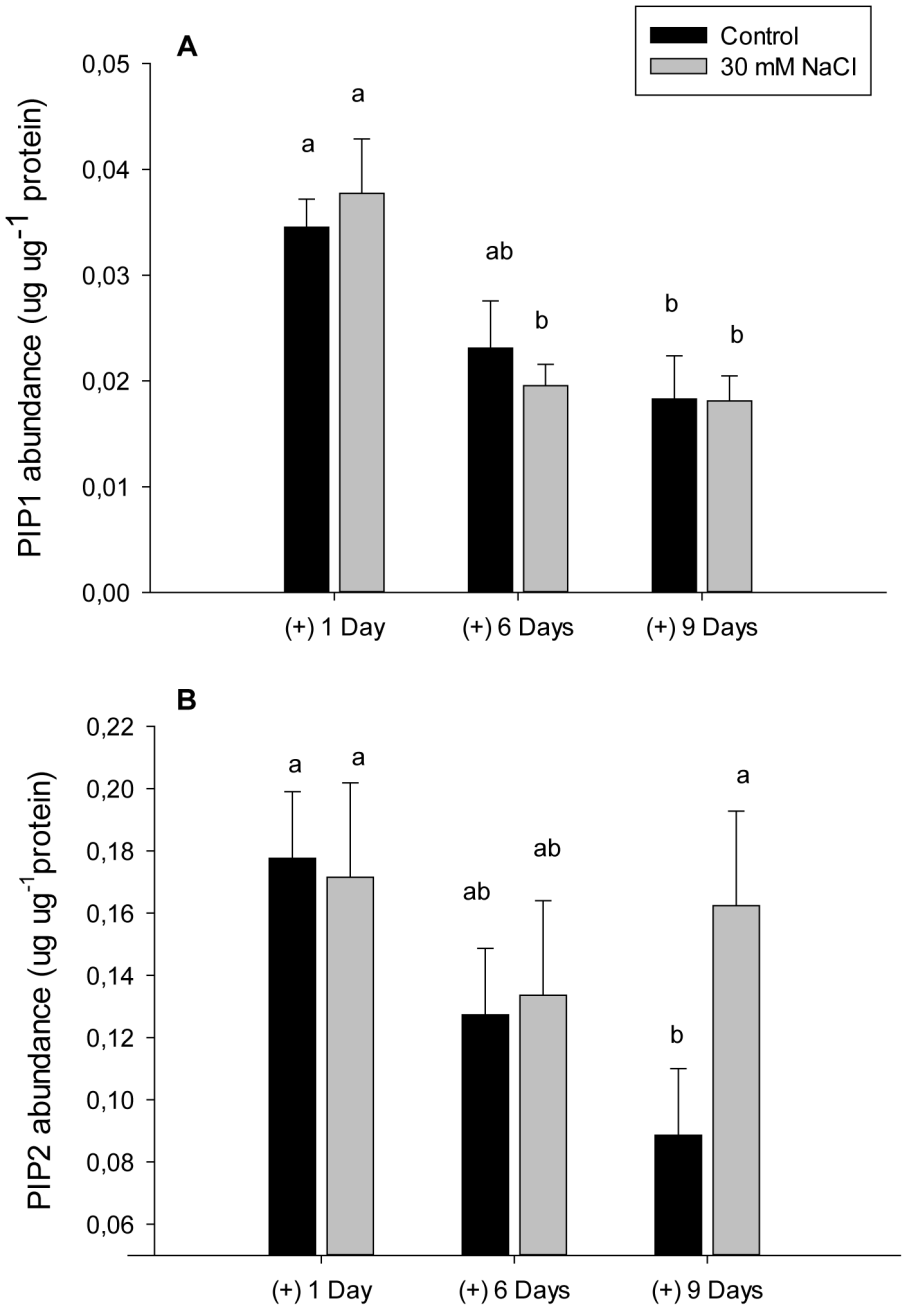
Protein abundance. PIP1 (A) and PIP2 (B) protein abundance in roots of *P.vulgaris* plants treated with 0 and 30 mM NaCl after 1, 6 and 9 days. PIP1 and PIP2 refer to the use of primary antibodies that recognize several PIP1 and PIP2 proteins respectively. Significant differences between treatment means at different days of measurement are shown with different letters at α = 0.05. Means (n = 3) ± SE are shown.

PIP1 protein abundance decreased after 9 days of treatment in both control and NaCl treated plants compared with day 1. However, there were no differences among control and NaCl plants when comparing the same day of measurement (Figure 3A). On the other hand, protein abundance analysis showed a significantly increase in PIP2 content after 9 days of NaCl treatment compared with control plants at the same day of measurement (Figure 3B). There were not significant differences in the abundance of PIP2 phosphorylated proteins among treatments at any of the measured days (data not shown). The data from the western blot analysis showed that the antibodies against phosphorylated PIP2 could recognize the same protein bands than the antibodies against non-phosphorylated PIP2 (Figure S1). The data from the cross-reaction analysis between antibodies against PIP2 and against phosphorylated PIP2A, PIP2B and PIP2C, determined the specificity of each antibody as they could recognize only their particular peptide (Table S2).

### Root PIP immunolocalization and suberization of cortical layers

**Figure 4:**
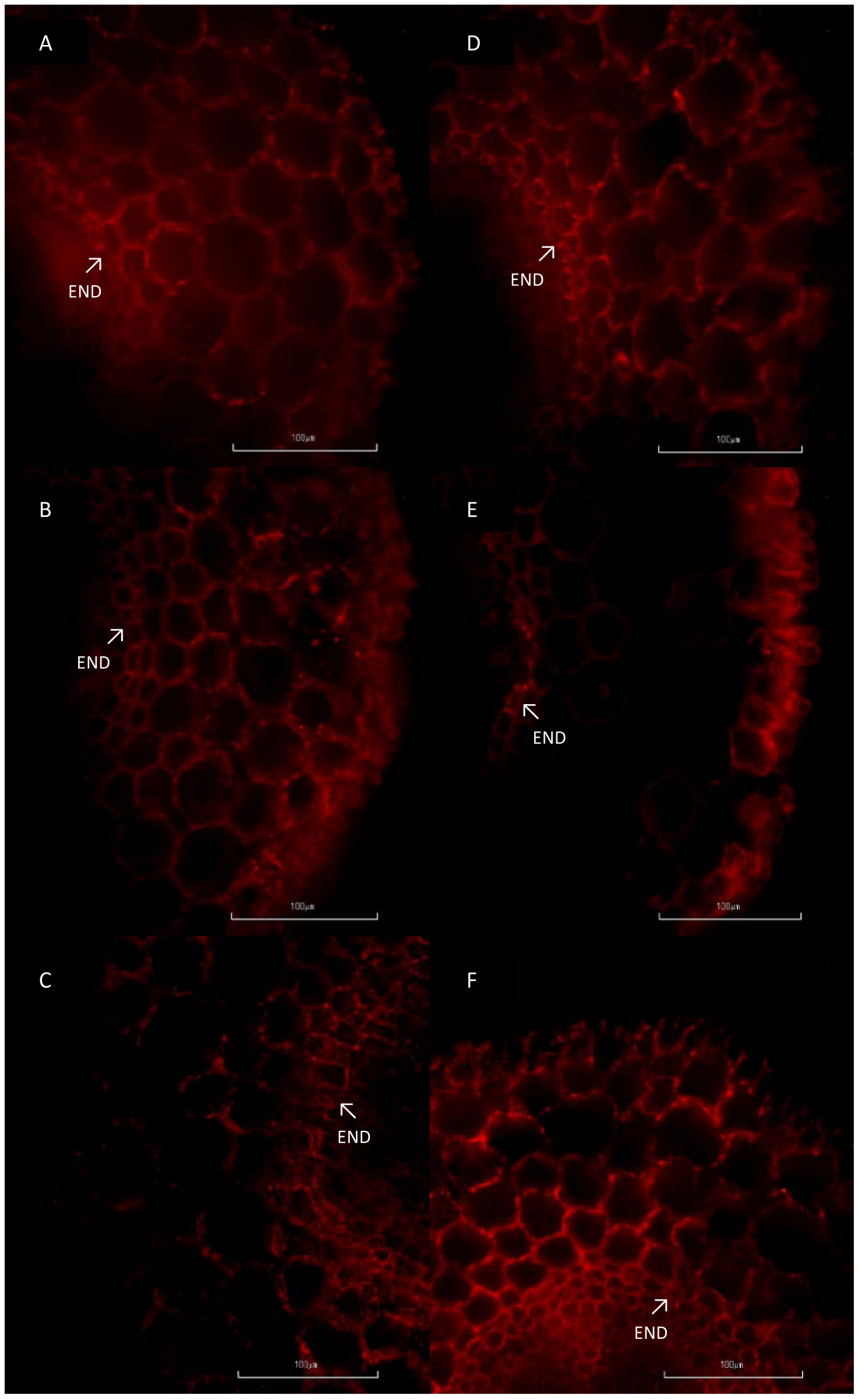
PIP2 immunolocalization. Immunolocalization of PIP2 aquaporins in cross sections of control (non-treated) plants (A–C) and salt-treated plants (D–F) after 1 (A, D), 6 (B, E) and 9 (C, F) days of treatment. The sections were taken at 0.5 to 1 cm from the root tip and examined under a fluorescent microscope with a red filter G-2A at 562 nm excitation and 576 nm emission. Endodermis (END) is indicated with arrows. Bar scale 100 µm.

PIP1 antibody showed similar immunostaining patterns in control and salt treated plants at the different days of measurements (data not shown), with low immunostaining signal although well distributed within the root cortex. PIP2 antibody showed the same immunostaning patter at day 1 in control and salt-treated plants, with homogenous signal and distribution within the root cortex (Figure 4A,D). Sections of roots after 6 days of treatment showed a different distribution between control and salt-treated plants. Sections from control seedlings showed homogenous distribution within the whole cortex, while plants affected by salt were mostly distributed in the outer part of the cortex close to the epidermis and also around the vascular cylinder (Figure 4B,E). Root sections after 9 days of salt treatment showed higher signal in salt-treated plants compared with controls plants, with a PIP2 signal evenly distributed within the root cortex (Figure 4C,F).

**Figure 5:**
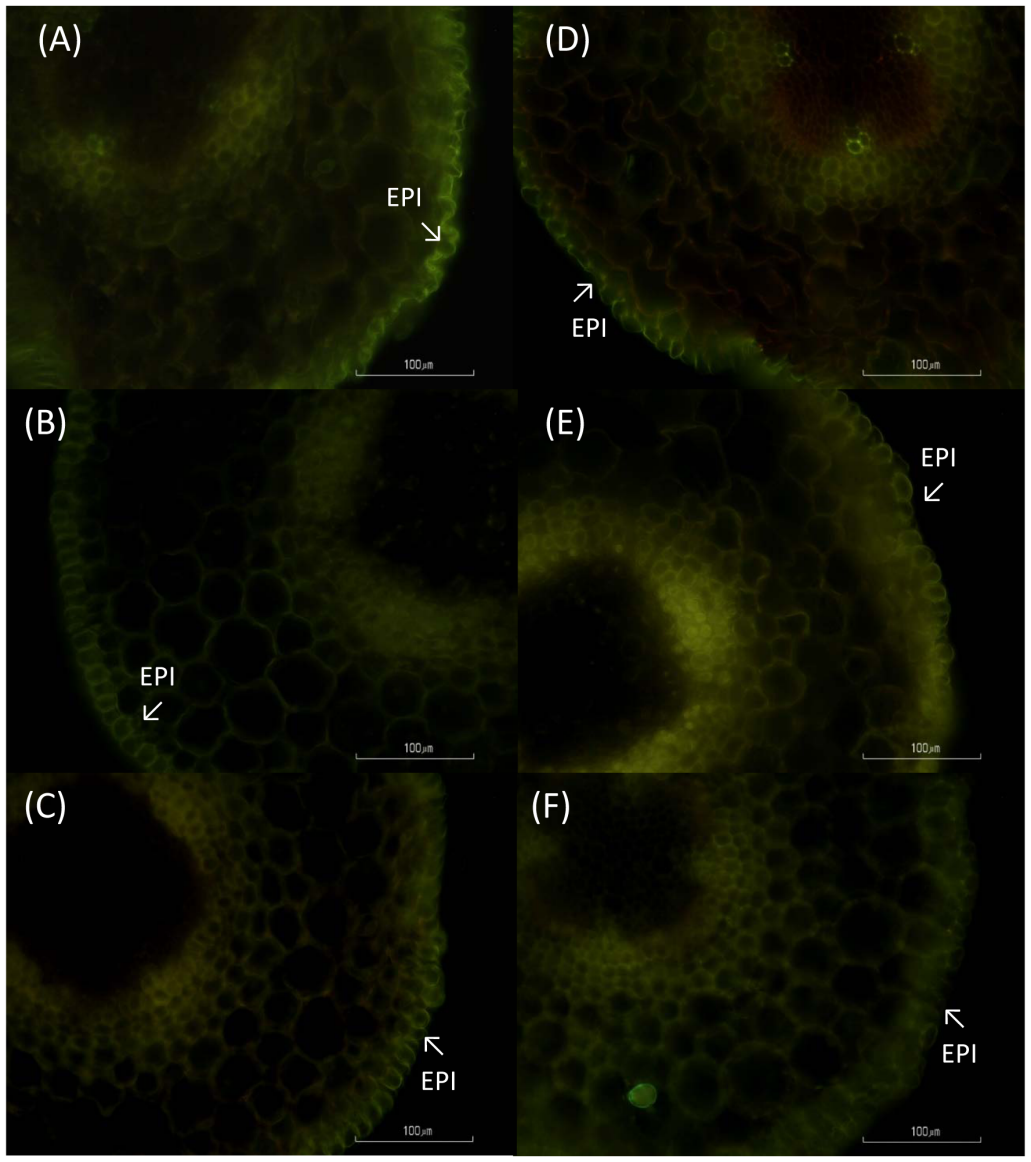
Root endodermal and exodermal suberin accumulation. Root cross sections of control (A–C) and NaCl treated roots (D–F) after 1 (A, D), 6 (B, E) and 9 (C, F) days. The sections were examined under the fluorescence microscope with a green light filter B-2A at 470–490 excitation and 505 emission. Epidermis (EPI) is indicated with arrows. Bar scale 100 µm.

Roots sections, both for control and salt treated plants, stained with berberine hemisulphate showed a poorly developed xylem, with the absence of casparian strips at the exodermis or the endodermis at any of the measured days (Figure 5), however both layers show some degree of suberization (Figure 5).

### Root metabolites determination and root damage

NaCl treatment did not cause any significant differences in root proline content, root electrolyte leakage nor oxidative damage to lipids at any of the measured days (Table S3).

Root glucose content in NaCl treated plants was lower at day 1 but did not show any significant differences after 6 and 9 days of treatment (Table 1). Root fructose content was significantly lower for NaCl plants after 1 day of treatment compared with control plants (Table 1). After 6 days of treatment, root fructose content in NaCl plants was higher than in controls ones, exceeding the values of day 1. After 9 days of treatment, root fructose of NaCl treated plants was similar to control plants, although still exceeding the values of day 1 (Table 1). Root sucrose content did not show any significant difference at any of the measured days in control and NaCl plants (Table 1).

**Figure 6:**
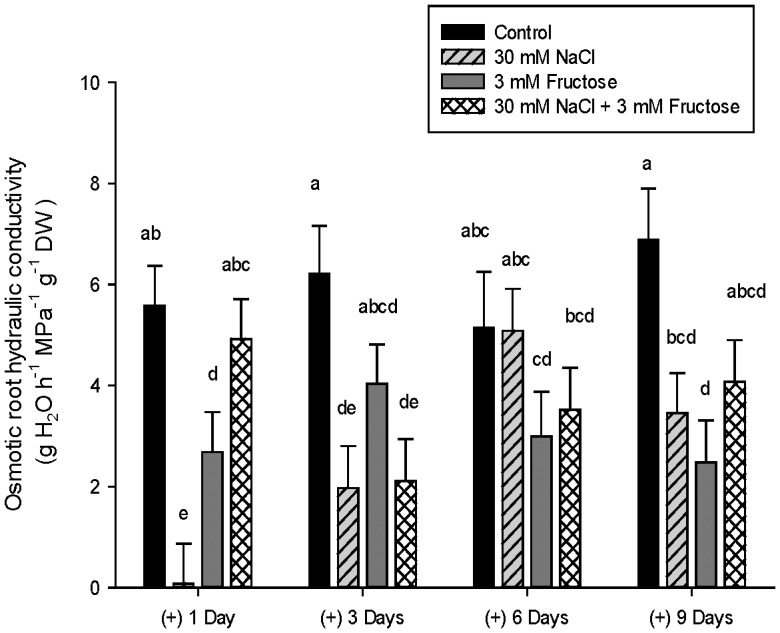
Root hydraulic conductivity with salt and fructose. Root hydraulic conductivity in *Phaseolus vulgaris* treated with of 0 (Control), 30 mM NaCl, 3 mM Fructose and 30 mM NaCl +3 mM Fructose after 1, 3, 6 and 9 days determined by the exudates method. Significant differences between treatment means at the different days of measurement are shown with different letters at α = 0.05. Means (n = 6) ± SE are shown.

As the results from the root fructose content coincide with a recovery of L in NaCl treated plants, we tested whether the presence of fructose within the solution may affect L in the presence or not of NaCl. The results showed, as in the previous experiments, an inhibition of L_o_ in NaCl treated plants after one day of treatment with a recovery at day 6 (Figure 6). Fructose treated plants had the same rates of L_o_ at all measured days. The combination of 3 mM fructose and 30 mM NaCl inhibited the reduction of L_o_ caused by NaCl treatment alone at day 1, but not at day 3 (Figure 6). Those plants treated with fructose recovered their L_o_ values after 6 days of treatment, being them inhibited again at day 9 (Figure 6).

### Na^+^ and Cl^−^ tissue concentrations and xylem sap content

Na^+^ accumulated mainly in the roots of plants with values twenty times higher than in the leaves after 6 and 9 days of treatment (Table 2). Cl^−^ accumulated mainly in the leaves, being five times higher in NaCl than in control plants after 9 days of treatment (Table 2).

**Table 2:** Sodium and chloride tissue and xylem sap concentrations.

	(+) 1 Day		(+) 6 Days		(+) 9 Days	
	Control	NaCl	Control	NaCl	Control	NaCl
Root Na^+^	1.78±0.13 c	9.49±0.64 b	2.72±1.77 c	13.07±1.14 a	1.00±0.08 c	12.46±0.87 ab
Leaf Na^+^	0.38±0.17 a	0.20±0.07 a	0.34±0.09 a	0.51±0.04 a	0.42±0.09 a	0.51±0.14 a
Root Cl^−^	3.82±0.43 b	6.64±0.72 a	2.77±0.51 b	6.23±1.57 a	1.77±0.11 b	5.84±0.46 a
Leaf Cl^−^	1.79±0.05 b	2.74±0.32 b	2.17±0.11 b	11.39±1.62 a	2.80±0.39 b	13.45±0.35 a
Xylem sap Na^+^	1.05±0.39c	2.67±1.08c	2.33±0.94c	22.32±10.42b	1.90±0.28c	50.64±6.17a
Xylem sap Cl^−^	12.89±0.83d	21.11±5.75c	15.54±5.39cd	67.63±22.78b	9.92±0.44d	132.89±13.81a

Root and leaf Na^+^ and Cl^−^ concentrations (mg g^−1^ DW) and Na^+^ and Cl^−^ xylem sap content (µg g^−1^ FW h^−1^) in *Phaseolus vulgaris* plants treated with 30 mM NaCl after 1, 6 and 9 days. Significant differences between treatment means at the different days of measurement are shown with different letters at α = 0.05. Means (n = 6) ± SE are shown.

The concentration of Na^+^ and Cl^−^ in the xylem sap was significantly higher after nine days of treatment compared with day one and six in NaCl treated plants. The amount of Cl^−^ transported within the xylem sap was higher than the amount of Na^+^ at all the days of measurement (Table 2).

## Discussion

We studied the effects of 30 mM NaCl in *Phaseolus vulgaris* plants, with a special focus on the parameters that affect root water transport. The applied NaCl treatment did not induce visual leaf damage, root electrolyte leakage, or peroxidation of lipids. These confirm that the amount of salt applied produced a moderate stress to plants and did not affect membrane integrity or cause any root or shoot damage due to the presence of Na^+^ and Cl^−^ in the tissues, besides a final growth inhibition.

Root hydraulic conductivity was determined by two methods with similar results. L_o_ only represents the water circulating by the cell-to-cell pathway, while L_p_ (determined with a pressure chamber) represents the total water circulating from the solution to the xylem vessels [12]. L_o_ and L_p_ have been previously found to follow the same pattern [51], [52], and, in our case, may be an indicator that the cell-to-cell is the main water transport pathway in beans.

Salt treatments affected plants by reducing their *gs*, L, and leaf water potential on day 1 and again after 9 days of treatment with a transient recovery after 6 days of treatment. The reduction of *gs* and L within the first hours of salt treatment have been usually observed, and it is one of the first symptoms of plants not being able to maintain their water balance [17], [18], [29], [31] [53]–[55], and a mechanism to avoid water loss through the stomata [3], [56]. These effects have been linked to a down-regulation of PIP gene expression [15], [16] and to the aquaporin phosphorylation/dephosphorylation state [17]. After 1 day of NaCl treatment, we only observed a reduction of the expression of *PvPIP2;2* aquaporin gene. This particular aquaporin did not show any water transport capacity when expressed in *Xenopus oocyt*es [57], so it is possible that the initial decrease of L was caused by membrane internalization containing PIP aquaporin proteins [15], [8].

The most novel finding of our research is the recovery of L after 6 days of exposure to NaCl. This recovery has been little documented with just a report of cell hydraulic conductivity increase over-time under salt stress in corn plants [19]. However, in our case, it could not be explained by the increase of the expression of the different PIP genes studied, protein abundance or phosphorylation state. We should consider that we analyzed the expression of just three PIP2 aquaporins (the only ones characterized until now in bean), and could be that other PIP2 genes, that the antibody recognizes, had a higher expression. Other possibility is that not always PIP gene expression and protein amount go in the same direction [49]. Furthermore, the studied phosphorylation state of the PIP2 proteins at their C-terminal tail could not have been ideal, as it has been documented that the aquaporins loop B also regulates aquaporin aperture [60]. Also, the effect of hormones accumulation, especially ABA, could have had a deep effect on the recovery of plants, as it has been previously shown to accumulate on beans under salt stress [58] and this accumulation may have been related with the increase of L [59].Nevertheless, inmunolocalization showed that PIP2 proteins where re-localized to cortical cells close to epidermis and cells surrounding xylem vessels. This redistribution of PIP2 proteins in the cortical cells could facilitate the entrance of water to the symplastic pathway, forcing the water to move mainly through the plasmodesmata or turned again to the apoplastic pathway. A recent study [61] has demonstrated that unsuberized parts of the roots have a higher root hydraulic conductivity and aquaporin expression than those suberized. According to this, we show a redistribution of PIP proteins at 5 to 10 mm from the root tip that may have caused the recovery of L in plants under salt stress. This is, as far as we know, the first time that this redistribution of PIP proteins under salt stress is described.

The recovery of L concurred in time with a recovery of root fructose content in salt treated roots. Sugars, especially glucose and sucrose, play an important role in stress alleviation through the regulation of plant osmotic potential [36], [62], [63], and as signal molecules [64]–[66], while maintaining the water status of the plants [36]. The knowledge of the role of fructose in plant functioning and development is less known. Cho & Yoo [66] found that high levels of fructose inhibited root growth, interacting positively with ABA signalling. Less studied is the role of fructose in plant water relations, although it has been shown to increase the exudation rates in sunflower plants [37], and we could demonstrate that the presence of fructose and salt in the solution can temporally maintain the root hydraulic conductance levels close to the control plants. In our study, we have shown that a higher accumulation of fructose within the root of salt treated plants happened at the same time of the recovery of L, although more information is needed to understand the role of fructose in plant water balance.

The down-regulation of *PvPIP2;1* gene expression after 9 days of treatment was related with a decrease in L and leaf water potential, and the increase of PIP2 protein abundance. The over-accumulation of PIP2 proteins was found not to be enough to recover L of chilled maize plants, and other mechanisms of stress injury avoidance were also needed [49]. In our study, the exposure to NaCl induced a reduction of growth that is quite common in plants under salt stress [11]. Also the xylem sap concentrations were higher in Na^+^ and Cl^−^ ions after 9 days of treatment and this effect would be one of the factors causing a decrease of leaf water potential and a reduction of *gs* and L.

In conclusion, our study has shown that *P.vulgaris* plants are able to overcome moderate levels of NaCl stress for a relatively short period of time by regulating their water balance. Plants were able to uphold their water balance by accumulating fructose, and redistributing the localization of PIP2 proteins in the root cortex. In response to a longer exposure to NaCl, there was reduction in growth, *gs* and leaf water potential that may have been one of that factors that diminished L.

## Supporting Information

Figure S1 **Western blot analysis.** Western blot analysis of microsomes from *Phaseolus vulgaris* roots. The antibodies used were against PIP2 and against phosphorylated PIP2A, PIP2B and PIP2C.
(PDF)

Table S1 **Alignment of aquaporins N and C-terminal regions.** Multiple alignment of N-terminal and C-terminal regions of *PvPIP1;1*, *PvPIP1;2* and *PvPIP2;1*, *PvPIP2;2* proteins, with *Phaseolus vulgaris PvPIP1;3* and *PvPIP2;1*, respectively. The consensus amino acids are underlined. The *PvPIP1;3* and *PvPIP2;1* sequences correspond to the peptide used to make the respective antibodies.
(PDF)

Table S2 **Antibodies cross-reaction analysis.** Cross-reaction analysis for PIP2, PIP2A, PIP2B and PIPC antibodies (horizontal) with their correspondent peptides (vertical). Bovine serum albumin (BSA) was included as a control reaction.
(PDF)

Table S3 **Root proline, ODL and EL.** Root proline (µmol g^−1^ DW), oxidative damage to lipids (ODL) (µmol g−1 DW) and root electrolyte leakage (EL) (%) of *Phaseolus vulgaris* control non-treated plants and plants treated with 30 mM NaCl after 1, 6 and 9 days. Significant differences among NaCl treatment means at the different days of measurement are shown with different letters at α = 0.05. Means (n = 6) ± SE are shown.
(PDF)
