# Supplementary material for: Mild Salt Stress Conditions Induce Different Responses in Root Hydraulic Conductivity of Phaseolus vulgaris Over-Time
Source: PLoS One. 2014 Mar 4;9(3):e90631. doi: 10.1371/journal.pone.0090631 (PMC3942473; doi:10.1371/journal.pone.0090631)

Figure S1. Western blot analysis of microsomes from *P. vulgaris* roots. The antibodies used were against PIP2 and against phosphorylated PIP2A, PIP2B and PIP2C.

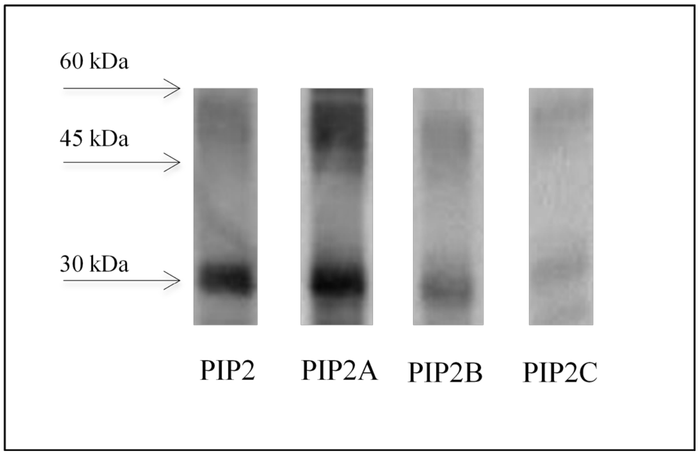

Supplement: Figure S1 — Western blot analysis. Western blot analysis of microsomes from Phaseolus vulgaris roots. The antibodies used were against PIP2 and against phosphorylated PIP2A, PIP2B and PIP2C. (PDF) [file pone.0090631.s001.pdf]
