# Supplementary material for: Mild Salt Stress Conditions Induce Different Responses in Root Hydraulic Conductivity of Phaseolus vulgaris Over-Time
Source: PLoS One. 2014 Mar 4;9(3):e90631. doi: 10.1371/journal.pone.0090631 (PMC3942473; doi:10.1371/journal.pone.0090631)
Supplement: Table S1 — Alignment of aquaporins N and C-terminal regions. Multiple alignment of N-terminal and C-terminal regions of PvPIP1;1, PvPIP1;2 and PvPIP2;1, PvPIP2;2 proteins, with Phaseolus vulgaris PvPIP1;3 and PvPIP2;1, respectively. The consensus amino acids are underlined. The PvPIP1;3 and PvPIP2;1 sequences correspond to the peptide used to make the respective antibodies. (PDF) [file pone.0090631.s002.pdf]

Table S1. Multiple alignment of N-terminal and C-terminal regions of *PvPIP1;1*, *PvPIP1;2* and *PvPIP2;1*, *PvPIP2;2* proteins, respectively, with *Phaseolus vulgaris* *PvPIP1;3* and *PvPIP2;1* respectively. The consensus amino acids are underlined. The *PvPIP1;3* and *PvPIP2;1* sequences correspond to the peptide used to make the respective antibody.

PvPIP1;3	MEGKEQDVSLGANKFSERQPIGTAAQ
PvPIP1;1	<u>MEGKEQDVSLGANK</u>YA<u>ERQPIGTAAQ</u>
PvPIP1;2	<u>ME</u>N<u>KE</u>E<u>DV</u>KV<u>GANKF</u>T<u>ERQP</u>L<u>GTAAQ</u>
PvPIP2;1	AIKALGSFRSNA
PvPIP2;2	<u>AIK</u>V<u>LGSFRSNA</u>
PvPIP2;3	<u>A</u>V<u>KALGSFRSN</u>P
