# Supplementary material for: Mild Salt Stress Conditions Induce Different Responses in Root Hydraulic Conductivity of Phaseolus vulgaris Over-Time
Source: PLoS One. 2014 Mar 4;9(3):e90631. doi: 10.1371/journal.pone.0090631 (PMC3942473; doi:10.1371/journal.pone.0090631)
Supplement: Table S2 — Antibodies cross-reaction analysis. Cross-reaction analysis for PIP2, PIP2A, PIP2B and PIPC antibodies (horizontal) with their correspondent peptides (vertical). Bovine serum albumin (BSA) was included as a control reaction. (PDF) [file pone.0090631.s003.pdf]

Table S2. Cross-reaction between PIP1, PIP2, PIP2A, PIP2B and PIPC antibodies (horizontal) with their correspondent peptides (vertical). Bovine serum albumin (BSA) was included as a control reaction.

	PIP2	PIP2A	PIP2B	PIP2C
PeptPIP2	**1.21**	0.11	0.07	0.06
PeptPIP2A	0.09	**1.02**	0.27	0.06
PeptPIP2B	0.08	0.17	**0.95**	0.06
PeptPIP2C	0.09	0.15	0.11	**0.12**
BSA	0.08	0.07	0.06	0.06
