# Supplementary material for: Mild Salt Stress Conditions Induce Different Responses in Root Hydraulic Conductivity of Phaseolus vulgaris Over-Time
Source: PLoS One. 2014 Mar 4;9(3):e90631. doi: 10.1371/journal.pone.0090631 (PMC3942473; doi:10.1371/journal.pone.0090631)
Supplement: Table S3 — Root proline, ODL and EL. Root proline (µmol g−1 DW), oxidative damage to lipids (ODL) (µmol g−1 DW) and root electrolyte leakage (EL) (%) of Phaseolus vulgaris control non-treated plants and plants treated with 30 mM NaCl after 1, 6 and 9 days. Significant differences among NaCl treatment means at the different days of measurement are shown with different letters at α = 0.05. Means (n = 6) ± SE are shown. (PDF) [file pone.0090631.s004.pdf]

Table S3. Root proline (µmol g^{-1} DW), oxidative damage to lipids (ODL) (µmol g^{-1} DW) and root electrolyte leakage (EL) (%) of *Phaseolus vulgaris* control non-treated plants and plants treated with 30 mM NaCl after one, six and nine days. Significant differences among treatment means at the different days of measurement are shown with different letters at α= 0.05. Means (n=6) ± SE are shown.

	(+) 1 Day		(+) 6 Days		(+) 9 Days	
	Control	NaCl	Control	NaCl	Control	NaCl
Root proline	0.7 ± 0.1a	1.0 ± 0.1a	0.7 ± 0.1a	0.7 ± 0.1a	1.0 ± 0.2a	1.3 ± 0.4a
Root ODL	0.1 ± 0.03a	0.2 ± 0.04a	0.1 ± 0.02a	0.1 ± 0.02a	0.2 ± 0.05a	0.2 ± 0.03a
Root EL	21.0 ± 0.8a	21.4 ± 0.6a	23.6 ± 0.9a	21.7 ± 0.9a	22.3 ± 0.9a	22.2 ± 0.7a
